# Duration of incapacity of work after tibial plateau fracture is affected by work intensity

**DOI:** 10.1186/s12891-018-2209-1

**Published:** 2018-08-07

**Authors:** Tobias M. Kraus, Charlotte Abele, Thomas Freude, Atesch Ateschrang, Ulrich Stöckle, Fabian M. Stuby, Steffen Schröter

**Affiliations:** 1 BG Trauma Center, Eberhard Karls University Tübingen, Schnarrenbergstr. 95, 72076 Tübingen, Germany; 2 Paracelsus University Salzburg, Landeskrankenhaus Salzburg, Salzburg, Austria

**Keywords:** Knee, Tibial plateau fracture, Professional activity, REFA, Return to work

## Abstract

**Background:**

Tibial plateau fractures requiring surgery are severe injuries of the lower extremity. Tibial plateau fractures have an impact not only on physically demanding jobs but notably on general professional life too. The aim of this study was to assess how the professional activity of patients will be affected after a tibial plateau fracture.

**Methods:**

39 consecutive patients (ages 20–61 years) were retrospectively included in the study and were clinically examined at a minimum of 14 month postoperatively. Inclusion criteria were surgical treatment of tibial plateau fractures between November 2009 and December 2012. The clinical evaluation included the Lysholm score and the Oxford Knee Score. Fractures were classified and analyzed using the AO classification. Intensity of work was classified as established by the REFA Association. The patients themselves provided postoperative duration of the incapacity of work and subjective ratings.

**Results:**

17 (43.6%) women and 22 (56.4%) men were examined with a mean follow-up of 29.7 ± 10.4 months (range 14–47). According to the AO classification there were 20 (51.3%) B-type-fractures and 19 (48.7%) C-type-fractures. The median incapacity of work was 120 days (range 10–700 days) with no significant differences between B- and C-type-fractures. Four (10.3%) patients reduced their working hours by 10.5 h per week on average. Patients with low workload (REFA 0–1, median incapacity of work 90 days, range 10–390 days) had a significant shorter incapacity of work than patients with heavy workload (REFA 2–4, median incapacity of work 180 days, range 90–700 days) (*p* < 0.05). The median Lysholm score decreased significantly from 100 points (range 69–100) before the injury to 73 points (range 23–100) at the time of the follow-up. All patients received postoperative physiotherapy (median 25 appointments, range 6–330), with a significant higher number of appointments for C-type-fractures than for B-type-fractures (*p* = 0.004).

**Conclusion:**

A relationship was found between workload and the duration of incapacity of work after tibial plateau fractures. The post-injury shift to less demanding jobs and the reduction of working hours highlight the impact of a tibial plateau fracture on a patient’s subsequent physical ability to work.

## Background

Tibial plateau fractures are severe joint injuries of the lower extremity. Since a notable number of patients sustaining tibial plateau fractures are young, active and in the middle of their working life, these injuries may have a profound effect on the individual’s professional career. The fracture patterns range from non-displaced split fractures and slightly or severely displaced depression fractures to complex comminuted fractures with severe destruction of the joint lines and cartilage lesions. Since the clinical outcome after tibial plateau fractures is closely related to the quality of the reduction [1, 2], each surgical technique must aim for an anatomic reconstruction of the injured joint. However, despite sophisticated understanding of the fracture patterns and modern anatomic angular stable implants the precise reconstruction of displaced or comminuted tibial plateau fractures can be challenging even for experienced trauma surgeons [3].

In the past surgeon oriented outcome measurements such as the Tegner-, Lysholm- or Oxford-Score were widely accepted and established as the only clinical outcome measurement in knee surgery [4, 5]. Other studies focused on return to sports after tibial plateau fractures [6, 7] but overtime patient-reported outcome measurements have gained importance as well as tools for assessing the return to daily activity and working life [8, 9].

However, so far no study has considered the sequelae after tibial plateau fractures and the impact on returning to work or subsequent work intensity. Therefore, the aim of this study was to determine the time until return to work and the professional capacity of patients after operative treatment of tibial plateau fractures. In particular, the study analyzed different work categories and different work intensities. The hypothesis was that heavy load workers would return to work later than white-collar workers.

## Methods

**Fig. 1:**
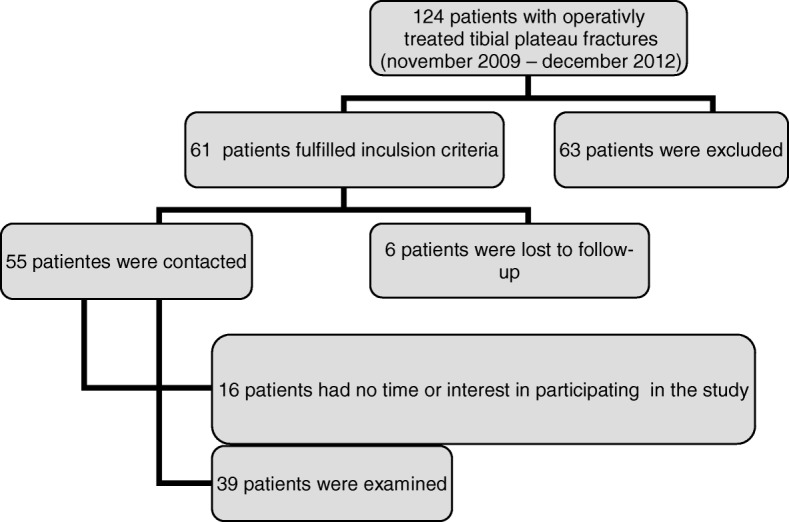
Patient flow chart

One hundred and twenty-four consecutive patients were treated surgically for tibial plateau fractures between November 2009 and December 2012 at a level I trauma center. Thirty –nine patients were included in this retrospective study. Inclusion criteria were surgically treated tibial plateau fractures, working capacity/employability at the time of the injury, age between 18 and 65 years (65 years is the normal date of retirement). Exclusion criteria were concomitant injuries of the same extremity (5), polytraumatized patients (9), posttraumatic conditions (3), age under 18 (4) / over 66 (42) – Fig. 1.

All fractures were classified according to the AO-classification (Arbeitsgemeinschaft Osteosynthese) [10].

### Clinical outcome and scores: Lysholm and Oxford Knee Score

The follow-up visit included a clinical examination, the assessment of the Lysholm score [4] and the Oxford-knee-score.

#### Work incapacity, REFA classification and reduction in earning capacity

A specific questionnaire was applied to gather data about the patient’s working live, work intensity, rehabilitation and sporting activity. The work intensity was classified according to the REFA Association (Table 1) [11–13]. To evaluate the health-related quality of life the SF-36-questionnaire was used.

**Table 1:** REFA classification of workload

Grade	Work intensity	Example
0	Work without special physical strain	Work without load like, for example, desk work
1	Work with small physical strain	Handling light work pieces; also lengthy standing or walking around
2	Work with moderate physical strain	Handling of 1–3 kg control device; carrying loads of 10–15 kg; climbing stairs or ladders without load
3	Work with hard physical strain	Carrying loads of 20–30 kg, shovelling, digging, chipping, climbing stairs or ladders with moderate load, moderate work in tense work posture
4	Work with most heavily physical strain	Carrying loads of more than 50 kg, climbing with heavy load, hard work in tense work posture

#### Radiological assessment

All fractures were assessed radiologically on plain radiographs of the knee in two planes. The severity of posttraumatic osteoarthritis was judged according to the score of Kellgren and Lawrence [14].

#### Surgical procedures

**Fig. 2:**
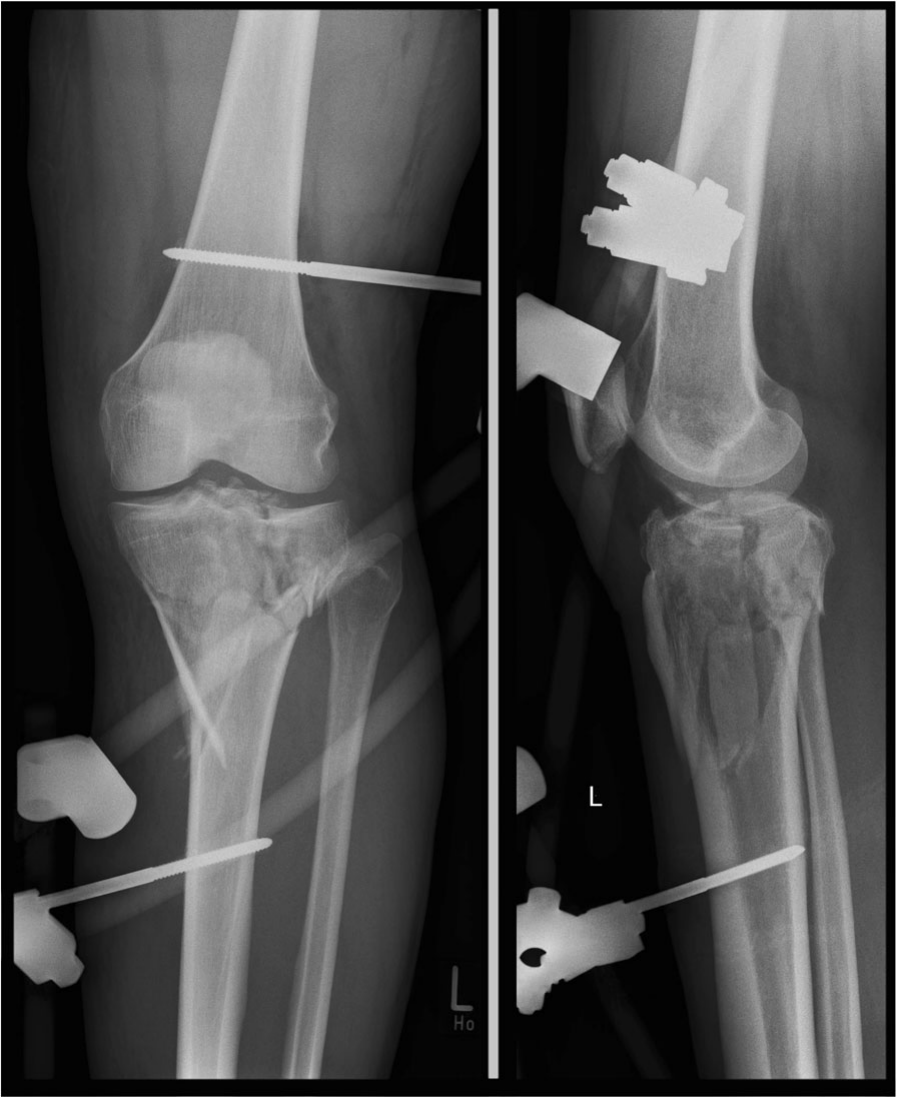
Massive destruction of the joint lines. Initial reposition in external fixator. AO 41 C3

**Fig. 3:**
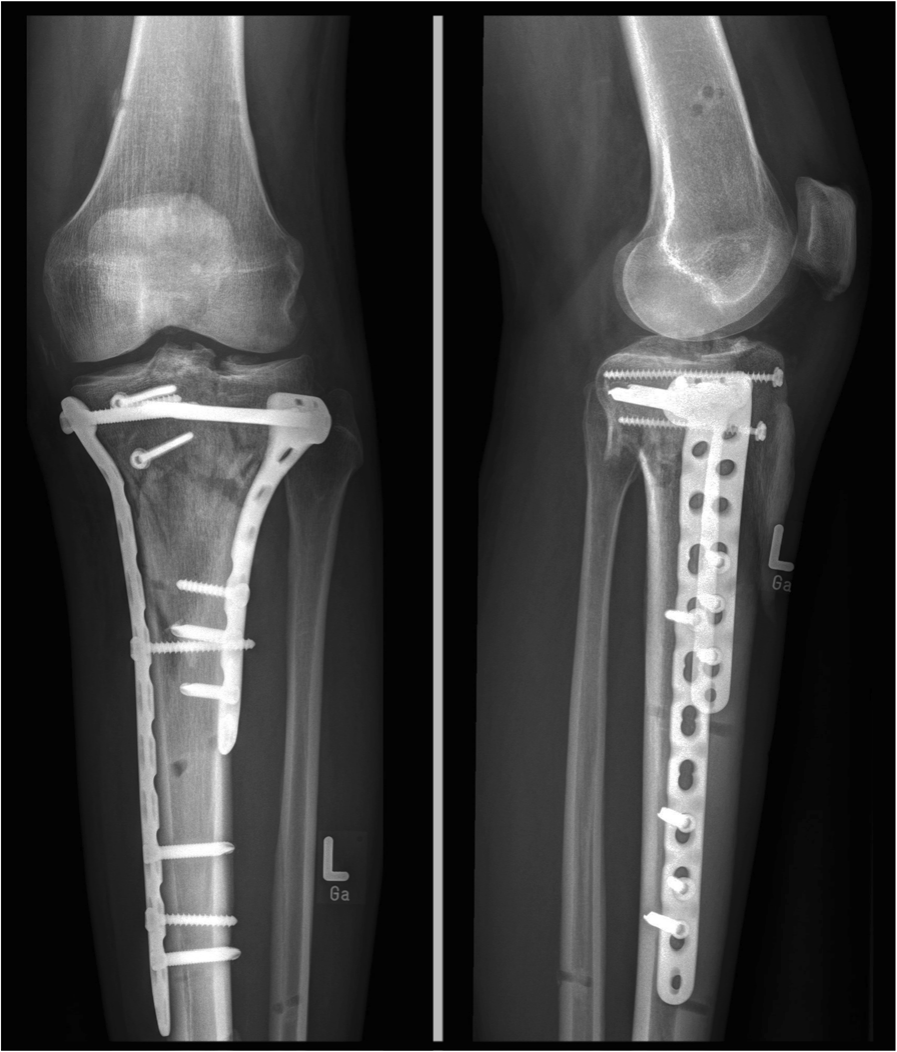
Double buttress plating and reconstruction of the joint lines

**Fig. 4:**
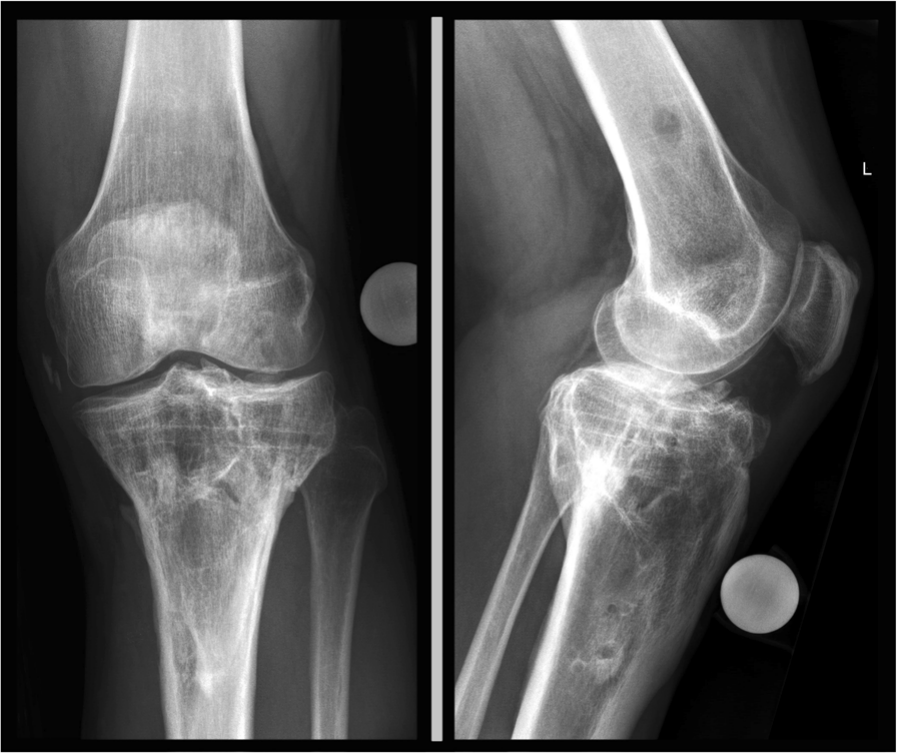
Long-term, 4 years follow-up with signs of osteoarthritis - Lysholm 89

**Fig. 5:**
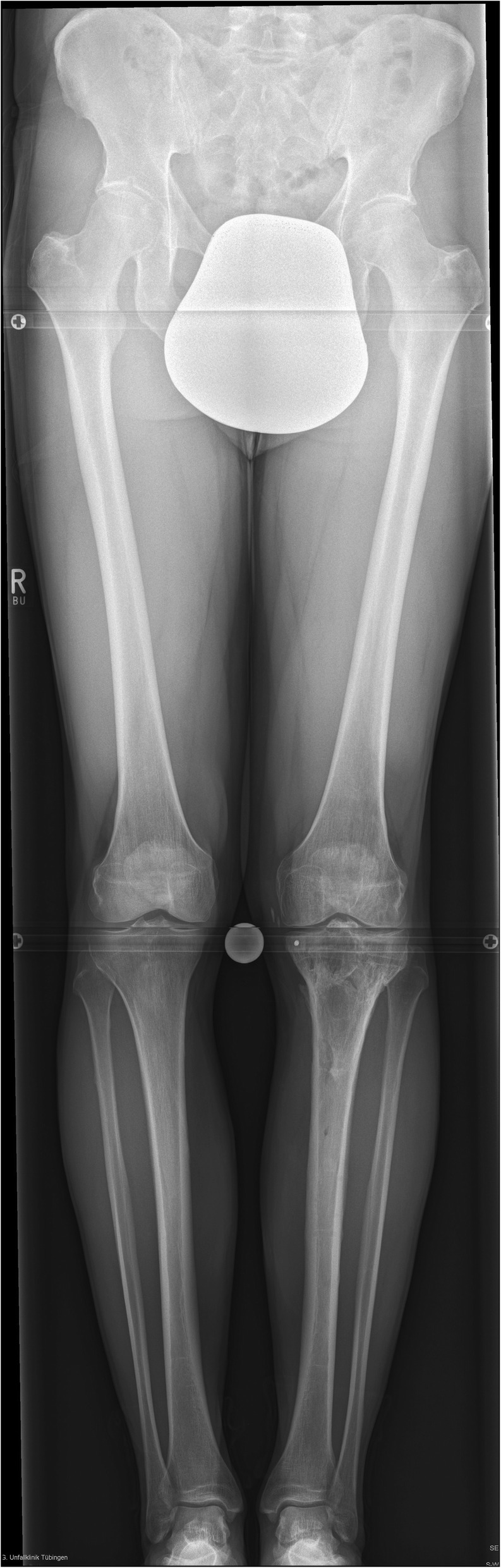
Long standing X-ray showing straight axis with no difference to contra-lateral side

The treatment of tibial plateau fractures in this study group was performed according to the AO principles [15–17]. CT scans are in most cases essential; in case of temporary external fixation, the CT scan is recommended to be performed after the external stabilization because with stretching of the fixateur an initial reposition is achievable [3]. Due to better visualization and understanding of the fractures themselves postero-lateral or postero-medial fragments were addressed as key fragments [16–18] (Figs. 2, 3, 4, and 5). Luo et al. have established a three-column fixation approach, especially useful for multiplanar fractures involving the posterior column [19], which was applied in this study group when necessary.

#### Post-operative protocol

Partial weight bearing is essential in the aftercare of tibial plateau fractures. The aftercare protocol was standardized and equal for all patients with 6 weeks of partial weight bearing.

#### Statistics

SPSS (Version 22, IBM Corp for Windows) was used for statistical analysis of the data. Correlation tests were performed using the Pearson and Spearman correlation coefficient and normally distributed results were compared using student’s t test. Non-normally distributed data were compared using the Mann-Whitney U test. All results are stated as mean ± standard deviation or median. The level of significance was presumed at *p* < 0.5. The statistical analysis was performed under guidance of the local institute for Clinical Epidemiology and Applied.

## Results

### Demographics

In total 39 patients were examined in the survey. All questionnaires were duly completed and all patients consented to the x-ray-examination. In 14 cases the treatment was performed in a two-stage procedure (external fixator / definite surgery). In all patients angular stable implants were used. Within the study group there were no infections, compartment syndromes or vascular injury.

The mean postoperative follow-up was 29.7 ± 10.4 months (range, 14–47). Our study group included 17 (43.6%) women and 22 (56.4%) men. The average age for both men and women at the time of the accident was 45.9 ± 10.1 years (range, 20–61).

27 (69.2%) fractures affected the left leg, 12 (30.8%) the right leg. According to the AO classification 51.3% of the fractures were B-type-fractures and 48.7% were C-type-fractures – Table 2.

**Table 2:** Fracture classification

AO classification	Frequency
B-type	20 (51.3%)
B1	2 (5.1%)
B2	5 (12.8%)
B3	13 (33.3%)
C-type	19 (48.7%)
C1	3 (7.7%)
C2	5 (12.8%)
C3	11 (28.2%)

### Causes of accident

The most common cause of tibial plateau fractures in the presented study group was sports accidents (41.0%), followed by low-energy-traumas (28.2%) such as falls from low height. Other common causes were traffic accidents (23.1%) and domestic accidents (7.7%). 13 (33.3%) fractures resulted from work-related accidents.

### Work incapacity, REFA classification and reduction in earning capacity

The median stay at hospital took 12 days (range 4–32) for the whole patient group. Patients with B-type-fractures (10 days, range 4–21) stayed a significantly shorter period than patients with C-type-fractures (20 days, range 5–32) (*p* = 0.034).

The median incapacity of work was 120 days (range 10–700) and there was no significant difference between B- and C-type-fractures. Four (10.3%) patients had to reduce their working hours by 10.5 h per week on average. Two patients retired after the rehabilitation due to the sustained tibial plateau fracture. Five patients had to reduce their work intensity, but four of them stayed within the same profession due to workplace modifications. One of these patients had to change his profession due to the functional impairment after the tibial plateau fracture.

According to the REFA classification patients (*n* = 23) with low work intensity (REFA 0 and 1) had a significantly shorter duration of work incapacity than patients (*n* = 13) with heavy work intensity (REFA 2–4) (Table 3). The longest duration of work incapacity was seen in the patients with C-type-fractures, who coincidentally were heavy load workers.

**Table 3:** Days until return to work

	Total (*n* = 39)		B-type-fractures (*n* = 20)		C-type-fractures (*n* = 19)	
	Mean	Standard deviation	Mean	Standard deviation	Mean	Standard deviation
Physical functioning	70.00	28.49	70.00	29.06	70.00	28.76
Physical role functioning	69.87	42.98	71.25	45.36	68.42	41.53
Bodily pain	63.15	26.62	63.80	27.73	62.47	26.13
General health perceptions	72.77	19.05	69.00	21.70	76.73	15.93
Vitality	53.59	17.54	55.00	20.00	52.10	14.93
Social role functioning	91.95	19.04	98.10	6.16	85.47	25.85
Emotional role functioning	82.05	37.35	80.00	38.09	84.21	37.46
Mental health	75.90	16.09	76.20	18.82	75.57	13.12

Reduction in earning capacity was noted in seven patients. The reduction in earning capacity was scored between 10 to 30% on average. The distribution of fracture types was equal between B (3 patients)- and C (4 patients)-type-fractures.

### Clinical outcome and scores

Thirty-one patients received postoperative physiotherapy (median 25 appointments, range 6–330). Eight patients were directly discharged to a rehabilitation clinic. There was a significant difference in the number of appointments between B- and C-type-fractures. The median of appointments for physiotherapy in patients with C-type-fractures was significantly more (50, range 10–330) than patients with B-type-fractures (18, range 6–56) (*p* = 0.004).

The patients were asked how physically fit they felt compared to the time before the fracture. At follow-up 72% of the patients felt physically less fit, 25,6% felt like having an equal level of physical fitness and only one patient felt fitter.

The median of the Lysholm Score decreased significantly from 100 (range 69–100) before the injury to 73 (range 23–100) at the time of the follow-up. Regarding the categories of the Lysholm score (excellent 91–100 points, good 84–90, fair 65–84, poor < 65) 92.3% of the patients had excellent results before the injury, whereas after the fracture 71.8% showed fair or poor results. In C-type-fractures a higher percentage showed fair or poor results (78.9%) compared to B-type-fractures (65.0%).

The median of the Oxford knee score (OKS) was 41 points (range 15–48) with no significant differences between B- and C-type-fractures.

All results for the SF-36 subscales were compared to the results of a standard population (Table 4). The results of 5 subscales for our study cohort were lower than for the standard population, the results of 3 subscales (general health perception, social role functioning, mental health) were higher. There were no significant differences in the results for B- and C-type-fractures.

**Table 4:** Detailed SF 36 subscale analysis

REFA classification	Number of patients	Minimum day off work	10%	25%	Median	75%	90%	Maximum day off work
0–1	23	10	29.4	63	90	150	216.8	390
2–4	13	90	102	130	180	377.5	590	700

### Radiological outcome

In 12 cases (30.8%) there was no difference in the x-ray assessment regarding osteoarthritis in comparison to contralateral. In 18 patients the injured knee joint was rated to be more affected by osteoarthritis compared to contralateral by one subscale according to Kellgren / Lawrence [13]. There was a difference by two subscales in eight patients (20.5%) and by three subscales in one case (2.6%).

## Discussion

The most important finding of this study was that incapacity of work was longer in the group with higher workload (median 180 days) compared to the group with low workloads (median 90 days). Given that we noted good mid-term results 29.7 months postoperatively (SD 10.4 months (range 14–47)), this cohort showed a good maintenance of knee function over time, particularly when considering that posttraumatic arthrofibrosis can often have a quick onset. However, a notable number (9 / 23.1%) of patients reported difficulties at their jobs forcing five employees to change to jobs with lower physical strains over time and forcing four patients to reduce the number of working hours per week (10.5 h/week). Although, despite there a relationship being found between the incapacity of work and workload, there was no such correlation concerning the fracture type. The median incapacity of work was 120 days (range 10–700 days) with no significant differences between B- and C-type-fractures.

In the literature several studies have already reported the outcomes after tibial plateau fractures (Table 5). The clinical results concerning the Lysholm Score 73.0 and Oxford Knee Score 37.3 ± 9.81 were in all subgroups comparable to those reported previously (Table 5).

**Table 5:** Outcomes of tibial plateau fractures

Author	Follow-up (in month)	Number of patients (*n*=)	Lysholm- Score (average scores)
Tscherne/Lobenhoffer (1993) [25]	70.8	190	78
Attmanspacher et al. (2002) [26]	48	41	84
Houben et al. (1997) [27]	61	46	84
Kraus et al. (2012) [6]	52.8	89	66.2
Siegler et al. (2011) [28]	59.5	21	86
Yu et al. (2009) [29]	23.7	54	79.5
Loibl et al. (2013) [7]	92	103	94.5
Müller et al. (2014) [30]	49	28	84.4
Rossbach et al. (2014) [21]	47.2	41	63.5
Van Dreumel et al. (2015) [31]	78.2	61	80.0 (KOOS)

In recent years patient reported outcome measures (PROMs) are gaining importance as these measures more reflect the satisfaction of patients after surgery rather than other outcome measures [20]. Just recently Baumann et al. reported on a study group of 77 skiers after tibial plateau fractures in a long-term follow-up study. It was found out that the PROM-score “forgotten knee score (FJS)”, which had been measured initially at arthroplasty, also significantly correlates with osteoarthritic radiologic knee joint degeneration in fracture cases [9].

However, to our knowledge there is no study that specifically considered return to work after sustained tibial plateau fractures.

Roßbach et al. recently examined patients after operatively treated tibial plateau fractures regarding the quality of life and the job performance. In that study polytraumatized patients and patients with other concomitant injuries of the same limb were included [21] making a comparison to other studies difficult. Eleven out of forty-one patients did not return to work, three patients had to change the profession after the injury after a follow-up of 47 month postoperatively.

Stevens et al. (2001) examined the outcome of 47 patients with operatively treated tibial plateau fractures with a mean follow-up of 8.3 years. They found similar results in the SF-36 scores for most of their patients under the age of forty compared to the healthy age-matched population. In the forty-and-over age group nine patients showed lower results in the SF-36 score compared to the healthy age-matched group. They found that the age of the patients seems to have more influence on the functional outcome rather than the type of the fracture and adequacy of reduction [22]. Also whereas the workload showed an effect on the incapacity of work in this study, age seems to be a minor factor for the clinical outcome. The subgroup analysis showed no differences in patients between 20 and 29 years, 30–45 years and patients between 46 and 65 years of age. We limited the inclusion to an age of 65 years of age because most employees retire at 65 years. This may create a certain bias in our study group as 42 elderly patients were excluded. So we cannot give any details about the recovery and final outcome of these patients. However, the average ages of the patients in this study group can be compared to the previously published studies.

In 2005 Litz et al. reported in his study significant differences in the results of the functional and radiological scores for the different types of tibial plateau fractures. Patients with C-type-fractures had worse results than patients with A- or B-type-fractures. Patients with C-type-fractures had a significant longer incapacity of work (40.5 weeks on average) than patients with A-type-fractures (21.1 weeks) and B-type-fractures (21.9 weeks) [23]. Yao et al. (2014) also found that C-type-fractures had the worst functional score results [24]. It would also be interesting to compare outcomes of infected osteosynthesis or patients with compartment syndrome in a long-term follow up. Fortunately we cannot provide any data, as none of the included patients sustained any of these complications. These complications were noted in the excluded polytraumatized patients.

There are also studies that report on the outcomes in sportsmen, including alpine skiers. Loibl et al. reported that overall only 49% of skiers returned to alpine slopes after a sustained tibial plateau fracture [7]. However, alpine skiing demands highest grades of physical exertion.

Similar results are reported by Kraus et al. in a study with 89 patients after tibial plateau fractures [6]. In this detailed study patients were asked about their sporting habits before, 1 year after injury and at 4.4 years after injury. Interestingly the hours of sports performance did not significantly shrink in the final follow-up. It was observed that patients continued to perform sports but on a less demanding level, shifting form high-impact sports to sports like Nordic walking or swimming.

In the context of knee surgery Schröter et al. studied specific impairments after high tibial osteotomy and also measured the time of return to work and the postoperative workload [12]. In this study the patients returned to work 87 days after surgery (median 87; range 14–450 days). Whether the earlier return to work is related to a better fitness and activity level of the patients undergoing elective surgery remains speculation. Also in that study patients in heavy workload groups needed more time for recovery. The Lysholm Score revealed values of 81.7 ± 12.7 that are similar values to the study group presented and to the studies as shown in Table 5.

In comparison to the upper extremity the time until return to work is longer for patients undergoing knee surgery. A recently published study describes return to work after arthroscopic Bankart repair after 2.06 month (95% CI 1.55–2.68) for jobs with low physical strains and 3.40 month (95% CI 2.70–4.24) for jobs with high physical strains [11].

Several limitations of this study should be considered. The rehabilitation program was only standardized in the first weeks post-surgery. Also an impaired proprioceptive function after successful tibial plateau reconstruction may have hindered return to heavy work. As we assessed operatively treated fractures only, conclusions regarding conservatively treated, possibly less severe fractures are therefore not possible. A further limitation is the small inclusion rate of only 31%. Furthermore the retrospective study design, the heterogeneous patient population and the variation in the length of the follow-ups are noted limitations. However, this study provides first data concerning the incapacity of work and the rehabilitation time for different work groups according to their intensity of work. With the improved anatomically pre-shaped implants and the improved understanding of the tibial plateau fractures future studies need to prove the presented data in a prospective and longitudinal manner.

## Conclusion

In this study, a relationship was found between work intensity and the duration of incapacity of work after surgically treated tibial plateau fractures. The post-injury shift to less demanding jobs and the reduction of working hours highlight the impact of a tibial plateau fracture on a patient’s physical ability to work.

The long rehabilitation periods may stimulate demand for intense and standardized rehabilitation programs, especially for high intensity workers.

## Abbreviations

AO: Arbeitsgemeinschaft Osteosynthese
REFA: “Reichsausschuß für Arbeitszeitermittlung”
